# 2323. Pediatric Infectious Diseases Outpatient Telehealth Services During the COVID-19 Pandemic: Single Center Experience

**DOI:** 10.1093/ofid/ofad500.1945

**Published:** 2023-11-27

**Authors:** Celisse Zabalo, Athanasios Tsalatsanis, Ambuj Kumar, Claudia M Espinosa, Amol Purandare, Carina A Rodriguez, Claudia Gaviria-Agudelo

**Affiliations:** University of South Florida, Tampa, Florida; University of South Florida, Tampa, Florida; Morsani College of Medicine, University of South Florida, Tampa, Florida; MORSANI COLLEGE OF MEDICINE PEDIATRICS, Tampa, Florida; University of South Florida, Tampa, Florida; University of South Florida, Tampa, Florida; University of South Florida Morsani College of Medicine, Tampa, Florida

## Abstract

**Background:**

Telehealth (TH) offers valuable opportunities to improve healthcare delivery that may translate to better health outcomes. Utilization of TH by pediatric subspecialty programs has been successful in inpatient settings. Limited data is available on its applicability to outpatient Pediatric Infectious Diseases during the pandemic. We aim to assess the differences between in person vs TH services in a pediatric infectious disease practice.

**Methods:**

This retrospective cohort study included patients aged 0 to 21 evaluated in a pediatric infectious disease clinic from 1/1/2019 – 12/31/2022 across 5 locations in Southwest Florida. Data was collected for new or established unduplicated patients seen in consultation for an acute, subacute, or chronic pediatric infectious disease condition. All encounters were characterized by in-person or TH modality. Clinic encounters related to research were excluded.

**Results:**

Altogether, 2,207 patients made 6,905 appointments. Of those appointments, 1,034 (15%) were TH visits, and 5,871 (85%) were in-person visits. New patient TH visits represented 17% vs 22% for in-person. There was a significant difference between the TH and in-person groups in mean age (10 years in TH vs 11 years in-person, p-value=0.01), race (Black 29% in TH vs 42% in-person, p< 0.001), ethnicity (Hispanic/Latino 34% in TH vs 23% in-person, p< 0.001), and type of insurance (Commercial 29% in TH vs 24% in-person, p< 0.001). There was no significant difference in gender (Female 49% in TH vs 47% in-person, p=0.217). The most frequent diagnoses are described in Figure 1. Physicians provided 77% of the TH visits vs 44% for the in-person visits. Cancellation/no-show rate was significantly lower for TH vs in-person visits (21% vs 27% respectively, p< 0.001). Mean traveled distance for in-person visits was 21 miles vs 30 miles avoided for TH encounters.

**Figure:**
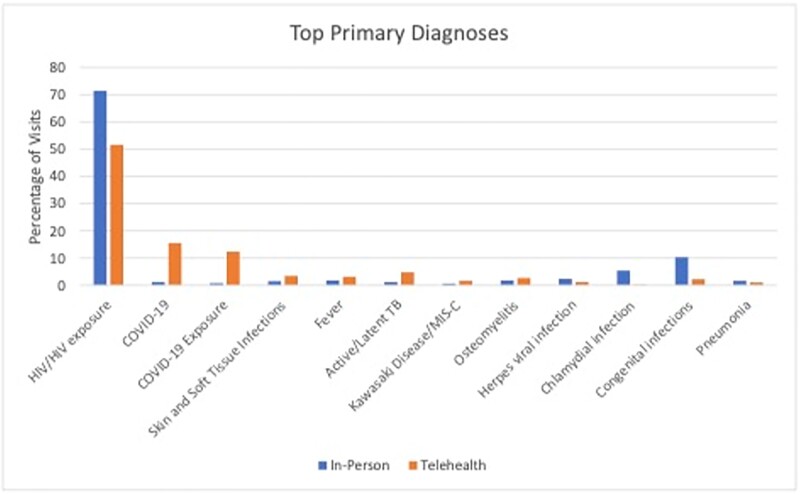

**Conclusion:**

TH offered a valuable option for pediatric infectious diseases patients during the COVID-19 pandemic. Availability of mobile connectivity, distance to appointment, and type of diagnosis may play a role in the selection of the appointment modality. TH holds promise for improving access to pediatric subspecialty services, allowing patient convenience and potential better health outcomes while delivering comparable care.

**Disclosures:**

**Claudia M. Espinosa, MD, MSc**, AstraZeneca: Grant/Research Support|Enanta: Grant/Research Support|Jansen and Jansen: Advisor/Consultant|Kentucky Rural Health Association: Honoraria|Merck: Grant/Research Support|Sanofi: Advisor/Consultant|Sanofi: Grant/Research Support|Sanofi: Honoraria|Sobi: Dinner **Amol Purandare, MD**, Caribou: Stocks/Bonds|Intellia: Stocks/Bonds|Merck: Grant/Research Support|Moderna: Grant/Research Support|Pfizer: Grant/Research Support **Carina A. Rodriguez, MD**, Gilead: Grant/Research Support|Moderna: Grant/Research Support|Novavax: Grant/Research Support

